# Diagnostic Values of Plasma, Fresh and Frozen Urine NT-proBNP in Heart Failure Patients

**DOI:** 10.5681/jcvtr.2014.024

**Published:** 2014-06-30

**Authors:** Mehrnoush Toufan, Hossein Namdar, Mohsen Abbasnezhad, Afshin Habibzadeh, Heidarali Esmaeili, Saeid Yaraghi, Zhila Samani

**Affiliations:** ^1^Cardiovascular Research Center, Tabriz University of Medical Sciences, Tabriz, Iran; ^2^Medical Philosophy and History Research Center, Tabriz University of Medical Sciences, Tabriz, Iran; ^3^Department of Pathology, Tabriz University of Medical Sciences, Tabriz, Iran

**Keywords:** Heart Failure, NT-proBNP, Fresh Urine, Frozen Urine, Plasma

## Abstract

***Introduction:*** The plasma N-terminal probrain natriuretic peptide (NT-proBNP) level is an important diagnostic and prognostic marker of heart failure. Recent studies have suggested urinary NT-proBNP as a new and simple test for diagnosis of heart failure. We aim to compare diagnostic value of plasma, fresh and frozen urine levels of N-terminal probrain natriuretic peptide (NT-proBNP) for detecting heart failure.

***Methods:*** Between January 2010 and January 2012, we measured urine and plasma levels of NTproBNP in 98 patients with chronic heart failure (CHF) and 29 age- and sex-matched healthy control subjects.

***Results:*** There were significant correlations between plasma NT-proBNP and fresh (r=0.45, p<0.001) and frozen (r=0.42, p<0.001) urine NT-proBNP concentrations in CHF patients. Due to receiver operating curve analysis, fresh and frozen urine NT-proBNP could diagnose HF with are aunder curve (AUC) of 0.73±0.04 (p<0.001) and 0.65±0.05 (p=0.01) with sensitivity and specificity of 73.97%, 58.62%, and 65.31%, 62.07%, for a cut-off of 94.2 and 96 pg/mL, respectively. Plasma NT-proBNP had greater AUC (0.94±0.02, p<0.001) and better sensitivity and specificity (94.9%, 89.66% for cut-off of 414.5 pg/mL). There was no significant correlation between LVEF and plasma, fresh and frozen urine NT-proBNP levels in CHF patients.

***Conclusion:*** Plasma NT-proBNP is still the best diagnostic marker with high sensitivity and specificity; however, urinary especially fresh urine NT-proBNP may be a surrogate to plasma NTproBNP for diagnosing HF with acceptable accuracy.

## Introduction

The incidence of heart failure (HF), as one of the most frequent causes of hospitalization in the general population, is increasing.^1,2^ HF is a disease that is characterized by poor prognosis and quality of life. Due to its socioeconomic burden, the early identification of HF and therapy in high risk patients is important. Besides history taking and physical examination in the evaluation of patients with HF, echocardiography is the most useful diagnostic test for HF.^1,3^

It is shown that natriuretic peptides are increased in HF; plasma B-type natriuretic peptide (BNP) and N-terminal propeptide of B-type natriuretic peptide (NTproBNP) levels have prognostic values for the diagnosis and prognosis of patients with suspected or established chronic heart failure (CHF).^4-8^ NT-proBNP has a long­er plasma half-life and higher plasma concentrations and so is of relevance for its use as a diagnostic tool.^8,9^ Although strong association is shown between HF and NT-proBNP, its clinical use is limited due to different reported values.^10-14^

Recently, assessment of the concentration of natriuretic peptides especially NT-proBNP in urine, as a non-invasive and simple test is suggested in CHF patients and several studies have evaluated its diagnostic and prognostic value.^15-21^ However, studies of urinary NT-proBNP are limited, particularly studies using fresh, unfrozen samples.^22^ This evaluation could be useful in certain circumstances.

We found only one study^22^ in the literature that have evaluated fresh urine NT-proBNP and compared it with plasma NT-proBNP levels. In this study we aim to evaluate and compare diagnostic value of plasma and fresh and frozen urine levels of NT-proBNP for detecting heart failure.

## Materials and methods

Between January 2010 and January 2012, 98 patients with CHF visiting our emergency department, Shahid Madani Heart Hospital, Tabriz, Iran were prospectively included in the study. Twenty-nine age- and gender- matched healthy subjects were also included. All patients had a history of chronic HF of at least 3 months’ duration and documented left ventricular impairment with a left ventricular ejection fraction (LVEF) <45%. The diagnosis of chronic HF was based on symptoms and clinical signs according to guidelines issued by the European Society of Cardiology^23^ and the American College of Cardiology.^1^ Patients with dyspnea of a non-cardiac-origin such as chronic obstructive pulmonary disease, bronchial asthma, pneumonia and anemia were excluded. Subjects with acute coronary syndromes, acute and chronic liver, pulmonary and renal diseases were also excluded. All patients gave written informed consent to participate in the study.

The following baseline clinical characteristics were prospectively recorded in detail on admission: age, sex, heart rhythm, bundle branch block, need for inotropic support, and current medication on hospital admission. Two-dimensional echocardiography was performed in all patients on arrival at emergency department, using a portable SonoSite Machine, US. The left ventricular ejection fraction was measured using the Simpson biplane method.

Blood and fresh urinary samples were collected for all patients on arrival at the emergency department. Venous blood was collected by venipuncture with the subject supine having rested quietly for at least 30 min. On the same day, blood samples and fresh spot urine samples were sent to the central laboratory immediately after collection. Blood was collected into a serum tube according to our local laboratory protocol. Another urine sample was collected into a standard urine collection tube without the addition of degradation inhibitors. After centrifugation at 2500 rpm and 4 °C for 10 minutes, urinary samples were separated and stored in cryotubes at -80 °C until assayed. Before the analysis, the urinary samples were centrifuged twice at 2500 rpm at 4 °C for 30 minutes to avoid possible NT-proBNP measurement interferences produced by the precipitation of salts in urine.

NT-proBNP measurements were performed in plasma and in urine on a Siemens 06606759 Immulite^®^ 2000 NT-proBNP, a commercially available electrochemiluminescent sandwich immunoassay (Cruinn Diagnostics, Mannheim, Germany). The analytical range was between 21.3 and 32855 pg/mL. Both investigators and patients were blinded to the NT-proBNP results.

### Data analysis

All data were analyzed using SPSS statistical package version 16.0 (SPSS Inc. Chicago, IL, USA). Continuous data with normal distribution are given as mean ± standard deviation, otherwise as median. Spearman correlation coefficients were calculated to determine the relationships between the urinary NT-proBNP concentration and plasma NT-proBNP and Left ventricle ejection fraction. Normally distributed values were evaluated with Student’s unpaired two-sided t-test. The Mann–Whitney U test was used for continuous variables. The receiver operating characteristic (ROC) curves were calculated and the area under the curves (AUC) and 95% confidence intervals were estimated as well as sensitivity, specificity. A *p *value less than 0.05 was considered significant.

## Results

In this study 98 CHF patients and 29 controls were included. Table 1  demonstrates baseline and laboratory characteristics between groups. Bundle branch block was observed in 23 (31.5%) of CHF patients including left bundle branch block in 16 and right bundle branch block in 7 cases. Electrocardiogram evaluation showed atrial fibrillation in 37 cases (37.7%) of CHF patients. CHF patients had significantly lower LVEF, higher systolic and diastolic blood pressure and higher BUN and serum creatinine levels.

**Table 1:** Baseline and laboratory characteristics in CHF patients and controls

	**CHF patients** **(n=98)**	**Controls** **(n=29)**	**P value**
Age (years)	62.52±12.49	58.57±16.24	0.12
Gender (male)	58 (59.2%)	19 (65.5%)	0.62
Weight (Kg)	70.84±12.34	72.51±11.36	0.52
Systolic blood pressure (mmHg)	130.01±23.56	116.00±13.70	0.003*
Diastolic blood pressure (mmHg)	77.87±13.35	71.57±9.41	0.02*
Left ventricle ejection fraction (%)	23.50±7.21	57.61±4.06	<0.001*
Sodium (mmol/L)	140.82±3.93	141.24±3.34	0.61
Potassium (mmol/L)	4.65±0.53	4.45±0.24	0.07
Blood urea nitrogen (mg/dL)	20.86±12.03	12.52±7.36	0.001*
Creatinine (mg/dL)	1.12±0.45	0.88±0.16	0.008*

* P is two-tailed significant.

Plasma, fresh and frozen urine levels of NT-proBNP were significantly higher in CHF patients (Table 2). Among CHF patients, we observed a significant correlation between plasma NT-proBNP and fresh urine NT-proBNP (r=0.45, p<0.001), between plasma NT-proBNP and frozen urine NT-proBNP (r=0.42, p<0.001) and between fresh urine NT-proBNP and frozen urine NT-proBNP (r=0.94, p<0.001).

**Table 2:** Comparison of mean plasma, frozen and fresh urine levels of NT-proBNP between CHF patients and controls.

	**CHF patients** **(n=98)**	**Controls** **(n=29)**	**P value**
Plasma NT-proBNP	9579.16±998.10	277.38±114.19	<0.001*
Fresh NT-proBNP	1707.62±592.21	118.56±27.39	<0.001*
Frozen NT-proBNP	1158.08±459.40	185.54±91.24	0.01*

* P is two-tailed significant.

**Figure 1:**
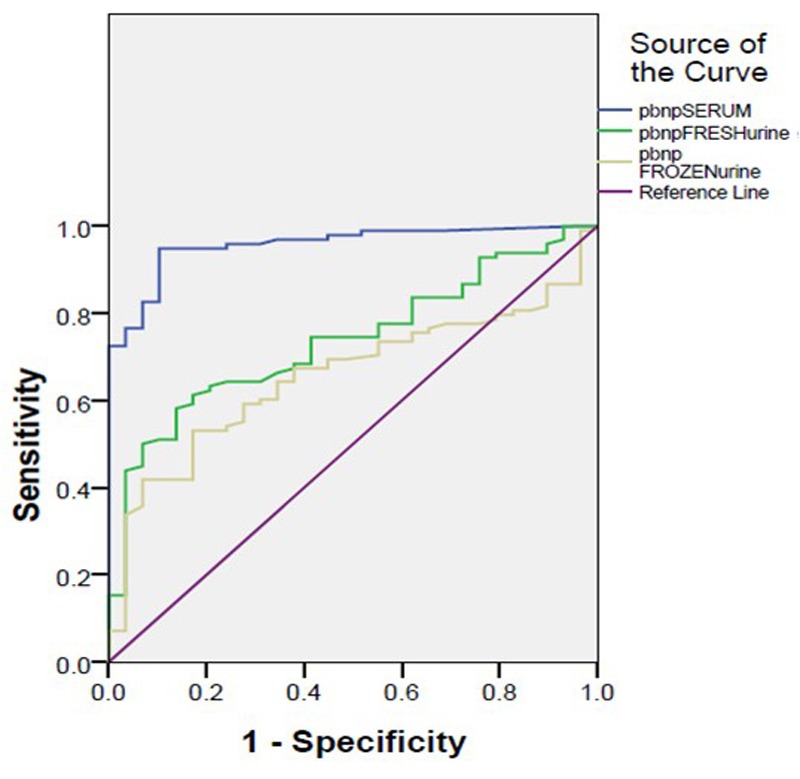
Receiver-operating characteristic curve analysis of plasma and urine NT-proBNP in obtaining CHF patients.

By creating ROC curves, we compared three NT-proBNP levels evaluation and identified a cut-off value for NT-proBNP that discriminates between patients with CHF and controls (Figure 1). Area under curve (AUC) ± SE was 0.95±0.01 (p<0.001) for plasma levels of NT-proBNP, 0.73±0.04 (p<0.001) for fresh urine levels of NT-proBNP and 0.65±0.05 (p=0.01) for frozen urine levels of NT-proBNP. Due to AUC, plasma NT-proBNP was more effective in diagnosing CHF patients. The evaluated cut-off points were 414.5, 94.2 and 96 pg/mL, for plasma, fresh urine, and frozen urine NT-proBNP respectively. According to estimated cut-off points, Sensitivity, specificity, positive and negative predictive value (PPV and NPV) and accuracy for plasma NT-proBNP levels was 93.81%, 86.67%, 95.78%, 81.25% and 91.12%, for fresh urine NT-proBNP levels was 71.13, 56.67, 84.14, 37.77 and 67.71%, and for frozen urine NT-proBNP was 63.92, 63.33, 84.93, 35.18 and 63.77%, respectively. In comparison of different AUC we observed that Plasma NT-proBNP has significantly higher and better AUC in comparison to Fresh (p=0.01) and frozen (p<0.001) urine NT-proBNP, but there was no difference between AUCs of fresh and frozen urine NT-proBNP (p=0.45).

We also divided LVEF levels in to LVEF<30% and 30 ≤LVEF <45% in CHF patients. There was no significant correlation between LVEF and plasma NT-proBNP levels (r=0.008, p=0.93), between LVEF and fresh urine NT-proBNP levels (r=0.05, p=0.63) and between LVEF and frozen urine NT-proBNP levels (r=0.05, p=0.64). AUC was calculated using ROC curve (The curve is not shown) to identify a cut-off value for plasma, fresh and frozen urine NT-proBNP levels in discriminating LVEF levels. The calculated AUC for plasma, fresh and frozen NT-proBNP levels were 0.50±0.06 (p=0.91), 0.40±0.06 (p=0.17) and 0.40±0.05 (p=0.15) with cut-off values of 4787, 143 and 131.5 pg/mL, respectively. The *p* value was not significant in any of them and so the sensitivity, specificity, PPV and NPV was not calculated for any of the NT-proBNP levels.

## Discussion

NT-proBNP is considered a useful marker in the diagnosis and management of heart failure. Recently, it is recommended that NT-proBNP in the urine could be used as a diagnostic marker. It gained more concern considering its availability and less invasive process. Previous studies showed that NT-proBNP is detectable in the urine of patients with HF and also in control, healthy subjects.^15-17^ There was also a good correlation between plasma and urine NT-proBNP concentrations.^17^ However, there are few studies evaluating urine levels of NT-proBNP and its accuracy in diagnosing CHF.^15-22^

In this study we evaluated accuracy of plasma, fresh and frozen levels of NT-proBNP in diagnosing CHF patients. To our knowledge, this is the first study on this field in our country. In this study we found good correlations between plasma and fresh and frozen urine NT-proBNP concentrations in CHF patients. In our study, all three measurements could discriminate CHF patients, but plasma NT-proBNP concentrations had the highest accuracy. The positive correlation between plasma and urine (fresh and frozen) NT-proBNP concentrations in CHF patients is demonstrated in previous studies with correlation coefficients ranging between r=0.53 and r=0.78.^15,17,18,21^ The report by Jungbauer et al.^22^ about fresh urine NT-proBNP showed a correlation coefficient 0.79. These were higher than evaluated correlations for fresh and frozen urine NT-proBNP in our study.

These findings are indicative of efficiency of urine and its use instead of plasma levels of NT-proBNP in diagnosing heart failure. However, in this study we found better accuracy for plasma than fresh and frozen urine NT-proBNP levels in diagnosing CHF with AUC of 0.95, 0.73 and 0.65, respectively. Similar to our findings, Michielsen and colleagues reported a significantly worse AUC for frozen urinary NT-proBNP measurement of 0.72, compared to 0.94 for plasma measurement.^19^ Unlike our findings, previous studies showed similar diagnostic value for plasma and fresh or frozen NT-proBNP levels.^16-18,22^ Due to higher AUC and relatively better sensitivity and NPV for fresh urine in comparison to frozen urine NT-proBNP concentrations, fresh urine measurements in the absence of plasma NT-proBNP evaluation, is more recommended.

The cut-off values for our evaluation was 414.5, 94.2 and 96 pg/mL for plasma, fresh urine, and frozen urine NT-proBNP, respectively, which is higher than other studies. Their mean levels were higher, as well. Because BNP correlates with age,^24^ the values were probably higher in our population as a whole, and thus the cut-off value was shifted upwards. Geographical variances in laboratory findings are reported variously and so the difference could be due to the characteristics of our population. We should also note that most of our CHF patients visited in emergency department with decompensated chronic heart failure, a state with worsening condition, which could cause increase in NT-proBNP concentrations. Similarly, Koç and colleagues^13^ with similar population of CHF patients but with lower mean age showed that with increase in severity of the CHF (NYHA class), the mean NT-proBNP increases. Another reason for the difference in the NT-proBNP values could be related to the fact that the clinical results of BNP and NT-proBNP assays are method-dependent.^25^ and so the analytical performance and clinical accuracy of any single immunoassay should be assessed in each laboratory.

Previous studies have also shown a significant correlation between plasma and urine NTproBNP and LVEF.^13,21,22,26^ Koç and colleagues^13^ showed that every 500-pg/mL increase in the concentration of plasma NT-proBNP was associated with a 14.2% increase in the risk of having LVEF < 30%. However, in our study we found no correlation between plasma and urine NT-proBNP and LVEF. It seems to be due to difference in patients group. Our patients were generally at decompensated state because of different causes, therefore the cardiac filling pressures and subsequently the NT-proBNP levels were increased in direct proportion with the degree of decompensation of HF, but not with the degree of left ventricular systolic dysfunction; So the NT-proBNP levels in plasma and urine correlate better with ventricular end diastolic pressures than LVEF, which was not evaluated in our study.

This study has some limitations; it was carried out in a single center that is a referral center for cardiology in Northwest Iran. It is possible that in general hospitals, the best cutoff value to detect CHF may be different. Moreover, all of patients entered in our study have been visited in emergency department showing that most of them were in decompensation. So we most probably cannot extend all the conclusions to the stable and compensated CHF patients. The exclusion of patients with comorbidities is another limitation, as in these patients the values would be higher and more diagnostic.

## Conclusion

In conclusion, the observed correlation between fresh and frozen urinary and plasma NT-proBNP is indicative of usefulness of urine levels for diagnosing HF. Both markers could be useful in clinical practice; however, plasma NT-proBNP is still the best diagnostic marker with high sensitivity and specificity and so urinary NT-proBNP should be used in the absence of plasma NT-proBNP evaluation for diagnosing HF. The slightly better predictive results for fresh urinary than frozen urinary NT-proBNP is encouraging to use fresh urine evaluations. Furthermore, due to insignificant slight correlations between LVEF and NT-proBNP levels among decompensated HF patients, the usefulness of this marker in diagnosing LVEF in these patients is questionable.

## Acknowledgments

This research was financially supported by Cardiovascular Research Center, Tabriz University of Medical Sciences, Iran. We had no external financial support. The authors have no conflicts of interest.

## Ethical issues

The project was approved by the local Ethics Committee and conducted in accordance with the guidelines of the Declaration of Helsinki.

## Competing interests

Authors declare no conflict of interest in this study.
